# Activation and desensitization of ionotropic glutamate receptors by selectively triggering pre-existing motions

**DOI:** 10.1016/j.neulet.2018.02.050

**Published:** 2019-05-01

**Authors:** James Krieger, Ji Young Lee, Ingo H. Greger, Ivet Bahar

**Affiliations:** a Department of Computational and Systems Biology, School of Medicine, University of Pittsburgh, 3501 Fifth Ave, Suite 3064 BST3, Pittsburgh, PA, 15260, United States; b Neurobiology Division, MRC Laboratory of Molecular Biology, Francis Crick Avenue, Cambridge Biomedical Campus, Cambridge, CB2 0QH, United Kingdom

**Keywords:** Ionotropic glutamate receptors, AMPA and NMDA receptors, Simulations, Molecular dynamics, Elastic network models, Allosteric interactions, Activation mechanism, Desensitization

## Abstract

Ionotropic glutamate receptors (iGluRs) are ligand-gated ion channels that are key players in synaptic transmission and plasticity. They are composed of four subunits, each containing four functional domains, the quaternary packing and collective structural dynamics of which are important determinants of their molecular mechanism of function. With the explosion of structural studies on different members of the family, including the structures of activated open channels, the mechanisms of action of these central signaling machines are now being elucidated. We review the current state of computational studies on two major members of the family, AMPA and NMDA receptors, with focus on molecular simulations and elastic network model analyses that have provided insights into the coupled movements of extracellular and transmembrane domains. We describe the newly emerging mechanisms of activation, allosteric signaling and desensitization, as mainly a selective triggering of pre-existing soft motions, as deduced from computational models and analyses that leverage structural data on intact AMPA and NMDA receptors in different states.

## Highlights

• Intrinsic motions reveal couplings between ionotropic glutamate receptor domains.
• Allosteric communication between domains selectively exploits pre-existing motions.
• Global dynamics facilitates activation, allosteric signaling and desensitization.
• Elastic network models efficiently elucidate the pre-existing dynamics of iGluRs.

## Graphical abstract

**Figure:**
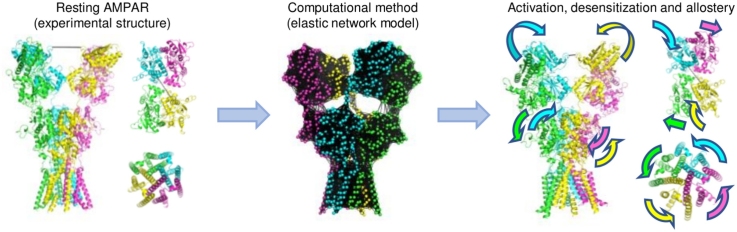

## 1 Introduction

In order to understand the brain, one needs to understand how its cells are connected (via synaptic junctions), the nature of the signals that are sent between them, and how these properties change over time for information processing and storage. These processes can only be understood in depth through a study of the molecular events involved in synaptic communication [1]. One key family of synaptic signaling molecules is the ionotropic glutamate receptors (iGluRs), which mostly reside on the post-synaptic side of a synapse. They open cation channels upon binding of pre-synaptically released glutamate to elicit various responses depending on their subunit composition, the pattern of pre-synaptic input, and their environmental conditions [[2], [3], [4], [5]]. There are four iGluR subfamilies two of which have been particularly well studied given their expression at most excitatory synapses and hence their predominant roles in synaptic transmission and plasticity: α-amino-3-hydroxy-5-methyl-4-isozazole propionic acid receptors (AMPARs), which respond on the millisecond timescale for moment-to-moment signaling [4] and initiation of synaptic plasticity [6], and N-methyl-d-aspartate receptors (NMDARs), which act as coincidence detectors that can pass much slower but larger currents enabling a calcium influx that drives synaptic plasticity in response to repetitive stimulation [3]. AMPARs also interact with a variety of other proteins that influence their trafficking, localization and function [[7], [8], [9]]. The two other iGluR subfamilies are the kainate receptors and delta iGluRs.

**Fig. 1:**
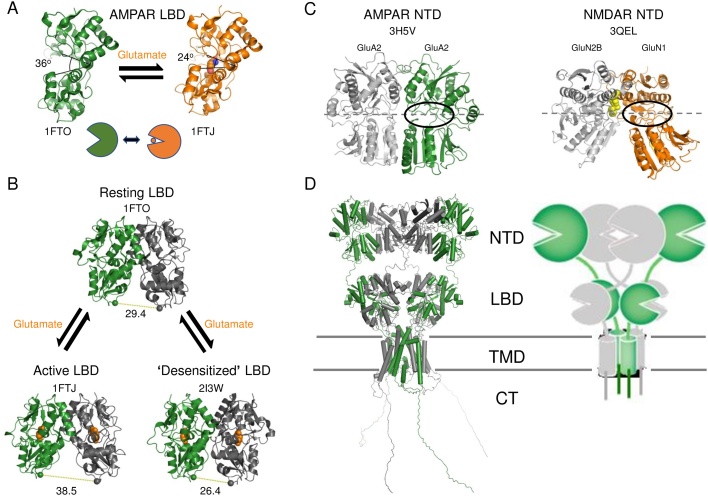
iGluRs are made of periplasmic binding protein (PBP) domains that close and undergo dimeric rearrangements. (A) The ligand-binding domain (LBD) is a clamshell domain that closes around the agonist glutamate. Shown are single subunits from LBD crystal structures in the ligand-free (apo) and glutamate-bound states together with their protein databank (PDB) codes. (B) LBDs form dimers with the two clamshells back to back. Closure of the two clefts pulls apart either the lower lobes to pull open the channel (activation) or the upper lobes to decouple from channel opening and remain closed (desensitization). (C) The NTD could also be produced as a separate module whose structure could be solved by X-ray crystallography. Two structures are shown representing the two main conformations and subfamilies. (D) A structure of the whole receptor is shown that is based on the resting state structure from cryo-EM (PDB 4UQJ), along with a schematic representation (on the *right*). Some linkers and the C-termini were modelled in with MODELLER.

A vast number of functional and structural studies have helped elucidate the molecular origin of channel responses observed in experiments, such as agonist-dependent channel opening to multiple conductance states and a process called desensitization where the channel closes with ligand still bound [8,10]. Structural studies effectively started once iGluRs were found to contain four glutamate binding sites in specialized ligand-binding domains (LBDs) that are structurally related to the clamshell-like periplasmic binding proteins (PBPs) found in bacteria and could be genetically excised and studied as separate modules [[11], [12], [13], [14], [15]]. The first structures showed that AMPAR LBDs indeed formed clamshells that close around ligands to drive channel opening akin to bacterial PBPs [16] (Fig. 1A).

The dimeric arrangement of the LBDs within a receptor, with two clamshells facing away from each other, gave rise to an initial model where cleft closure could either drive channel opening by pulling apart the lower lobes (Fig. 1B, left) or lead to desensitization by destabilizing the upper lobe dimer interface and decoupling cleft closure from channel opening (Fig. 1B, right) [16,17]. Structures of whole receptors revealed a complex multi-layered architecture with three structured domain layers (Fig. 1D) – the NTD, LBD and pore-forming transmembrane (TM) domain (TMD) – in addition to an unstructured C-terminal domain (CTD, which has not been resolved structurally to date) [[18], [19], [20], [21]]. The N-terminal domain (NTD) also belongs to the PBP superfamily and occupies different dimeric conformations (Fig. 1C) [[22], [23], [24], [25], [26], [27]], which enable it to allosterically regulate the channel in NMDARs [28,29].

In recent years, a number of agonist-bound crystal structures of whole AMPARs revealed some conformational changes without channel opening [[30], [31], [32]] and a cryo-electron microscopy study provided insights into activation and desensitization but could not clearly resolve whether and how the channel had opened [33], and the gating mechanism remained unclear. The situation has improved with recent structural characterization of auxiliary subunit complexes in resting [34,35], desensitized [36,37] and active states [36,38], as well as NMDAR structures in allosterically inhibited [39,40] and antagonist- and agonist-bound [27,41] states, but there are still a number of unanswered questions [42].

Structural data alone are not sufficient to make inferences on the mechanisms of function. Yet, the multitude of structures in different states have helped elucidate the conformational landscape accessible to these receptors. These, along with single-molecule experiments such as fluorescence resonance energy transfer (FRET) and related methods, nuclear magnetic resonance (NMR) spectroscopy, and double electron–electron resonance (DEER) spectroscopy have been applied to the study of iGluR dynamics [[43], [44], [45], [46], [47], [48], [49], [50], [51], [52], [53], [54], [55]]. Structural data have also opened the way to several structure-based computations, at various levels of resolution. Many questions regarding the activation and desensitization mechanism of iGluRs, as well as the allosteric events triggered upon ligand binding can now be explored by computational modelling and simulations of structural dynamics [[56], [57], [58]]. We provide below an overview of two major group of computations, molecular dynamics (MD) simulations and elastic network model (ENM) analyses for predicting structure-encoded dynamics, and the emerging mechanisms of function.

## 2 Computational methods for modelling protein dynamics

**Fig. 2:**
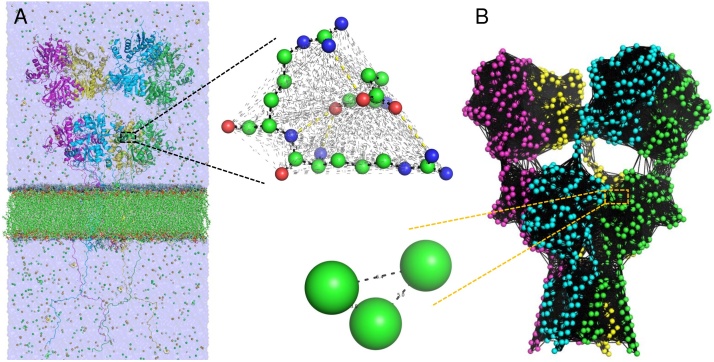
Two main methods for computational analysis of protein dynamics are illustrated. (A) All-atom molecular dynamics (MD) simulations include all atoms from the protein as well as surrounding water, salt and membrane. Shown is another model of a whole GluA2 AMPAR homotetramer including the C-terminal tails, generated using MODELLER and the CHARMM-GUI (785,175 atoms, of which 54,176 belong to the protein). The inset shows a zoomed view of three amino acids that are interacting and the myriad interactions between all their atoms. Covalent bonds are indicated by *thick black dashed lines*, Coulomb electrostatic interactions are *yellow* and van der Waals contacts are *grey*. **(B)** An elastic network model (ENM) representation is shown for the same AMPAR model with the C-tails removed. It has a node for each residue (a total of 3168 nodes). Uniform springs connect residues within 15 Å (*thick black lines*). The three residues shown in panel A are now represented by three nodes with springs describing their interactions, which oscillate around the starting distances shown.

Molecular dynamics (MD) simulations use a detailed force field to describe all the covalent and non-covalent interactions between all atoms in the system including the protein as well as surrounding water and ions (Fig. 2A). This method is based on a large number of time steps involving cycles of calculating all forces on all atoms and numerically integrating Newton’s equations of motion to update their positions and velocities, generating trajectories with full atomic detail [59]. The requirement of calculating the forces on all atoms every few femtoseconds [59] means that such methods are rather slow and can barely access time scales relevant for biology. They are indeed usually limited to hundreds of nanoseconds (ns) or less, especially for large systems such as multimeric receptors in membranes (785,175 atoms for the AMPAR-membrane-water system shown in Fig. 2A, made using MODELLER [60,61] and CHARMM-GUI [62]). The capabilities of MD simulations have somewhat improved with the development of specialized supercomputers [63] and with enhanced sampling schemes [64] that made it possible to calculate free energy landscapes associated with particular conformational changes and binding processes for small systems such as iGluR LBDs [65,66].

An alternative and complementary approach is to use a coarse-grained model, such as elastic network models (ENMs), which enables one to efficiently visualize the large collective modes of motions of the system at the expense of atomic details [67]. The adoption of ENMs in normal mode analysis (NMA) greatly speeds up the calculations, permitting one to evaluate the dynamics of whole iGluRs within minutes (real time) even in the presence of membrane. The most commonly used ENM is the anisotropic network model (ANM) [[68], [69], [70]]. Therein each residue is treated as a bead/node located at the position of the α-carbon and all interactions within a cutoff distance (of 12–15 Å) are modelled as uniform springs (Fig. 2B). The ANM provides a simple analytical expression for the Hessian matrix **H** of the second derivatives of the potential with respect to deformations. The decomposition of **H** yields a spectrum of normal modes of motions. The lowest frequency modes among them describe the most accessible collective motions that are usually functional, and occur at biologically relevant (microsecond to millisecond) timescales [71]. ENMs can also be used to explore allosteric signaling [72,73] and to identify important residues such as hinge sites and kinetic hotspots. The latter can be identified using the earlier and even faster Gaussian network model (GNM), which is more accurate at the expense of 3D information [[74], [75], [76], [77]].

## 3 Dynamics of iGluR domains revealed by MD simulations

The arrival of the first LBD structures from AMPAR subunit GluA2 in 1998 [78] and 2000 [11] was immediately followed by a number of MD simulation studies on this domain that continue to this day. Early studies were limited by computational power and therefore focused on small systems and short timescales. The main highlights of this period include: 1) simulations lasting 5 ns or less of LBD monomers in the presence and absence of agonists, which found that the open apo form showed larger fluctuations in line with the idea that agonists stabilized closed cleft conformations [79,80]; 2) MD simulations where kinetic energy was applied to specific binding site residues based on quantum mechanical calculations of vibrational energy transfer from agonist [81,82] and this energy was found to propagate through the LBD to dimer interface helix J where it would impact activation and desensitization [83]; and 3) the first enhanced sampling simulations of the LBD using a “partial template forcing” that drove large scale cleft opening and enabled analysis of associated atomic rearrangements and ligand dissociation [84,85].

Since then, studies have increasingly accessed longer timescales and carried out more detailed characterizations of conformational changes. This phase started around 2006 with simulations of the GluA2 LBD [86] and the GluN1 NMDAR LBD [87] that captured cleft closure in 20 ns of conventional MD. These were immediately followed by studies that used umbrella sampling for exploring cleft closure energetics, which found that the apo LBD was preferentially in the open state with a free energy difference of ∼4 kcal/mol with respect to the closed state [65,66]; the antagonist DNQX stabilized the open state; whereas the agonist glutamate stabilizes the closed state [65]. Similar analyses carried out on a larger panel of ligands showed that both binding and cleft closure energetics were important for efficacy [88] and that NMDA receptor LBDs showed a conformational selection mechanism that was not seen for the GluA2 AMPAR LBD [89,90].

Recent increases in computing power have further enabled the simulations of larger systems such as LBD dimers, which provided insights into rearrangements linked to desensitization [91,92], and NTD dimers, which revealed clamshell motions and interprotomer rearrangements akin to LBDs and metabotropic glutamate receptor ligand-binding cores, which may explain their allosteric signaling function in NMDARs [93,94].

Once whole receptor structures became available, simulations were carried out on tetramers including both the LBD and TMD for the GluA2 AMPAR [95] and the GluN1/2B NMDAR heteromer [96,97]. These were performed using targeted MD protocols to drive LBD conformational changes related to activation and desensitization, and the response of the TMD and the connecting linkers was studied. These studies indicated potential mechanisms whereby the linkers enable the LBDs to pull open the TMD channel in a subunit-specific manner.

Most recently, a set of MD simulations each on the order of five microseconds carried out with the Anton supercomputer for AMPAR LBD monomers and dimers revealed the detailed mechanism, energetics and kinetics of glutamate binding to individual LBD clamshells [98].

## 4 Dynamics of intact AMPAR and NMDAR revealed by elastic network models

**Fig. 3:**
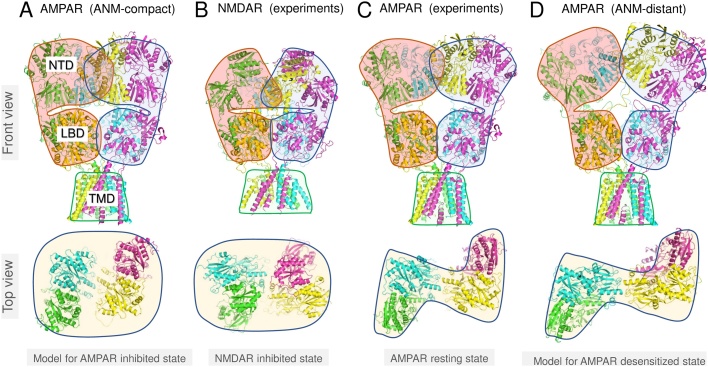
Global dynamics of iGluRs related to allosteric inhibition and desensitization. (A) A conformation generated by traversing ANM mode 4 of the original AMPAR crystal structure (PDB 3KG2) in one direction shows a compact packing of the NTD and LBD, which is highlighted in red and blue for the two pairs of NTD dimers and LBD dimers. The lower diagram displays the relative positions of the NTD dimers, which reveal a compact O shape when viewed from the extracellular region. (B) A similar compaction of the NTD and LBD is observed in the allosterically inhibited NMDAR crystal structure (PDB 4PE5) and a similarly compacted NTD arrangement is seen in the top view. (C-D) Traversing this ANM mode back in the other direction, we pass through the starting structure **(**C) where the NTD forms the classical N shape, and then reach an alternative conformation with the NTD dimers lifted away from the LBD and tilted away from each other and from the central symmetry axis similar to low resolution desensitized structures (D). See Movie 1 for more details, including cross-sectional views of the NTD, LBD and TMD layers.

**Fig. 4:**
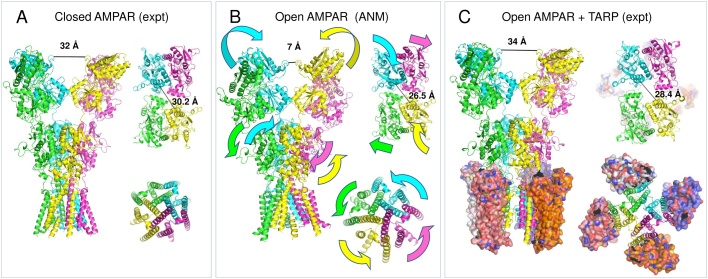
Global dynamics of iGluRs related to activation. (A-B) Comparison of the original AMPAR structure (PDB 3KG2) (A) and ANM intermediate along mode 6 (B) shows an iris-like rotation and opening of the TMD and NTD inter-dimer pivoting coupled to complex LBD dynamics, involving a rolling of the LBD dimers towards and away from each other together with a sliding rotation past each other. Top views of the LBD and TMD are displayed in each panel, along with color-coded arrows to more clearly show the collective motions of the four subunits and three layers (NTD, LBD and TMD) in mode 6. The latter can be viewed more clearly in Movie 2. (C) The TARP-associated open channel state (PBD 5WEO) reveals similar motions of the LBD and TMD but minimal NTD dynamics as a result of the modified NTD-LBD linker present in the crystallography construct used. The TARPs are shown in surface representation to highlight their optimized positioning for modulation of LBD and TMD dynamics. Black surfaces on the TARPs in the top view of the TMD layer indicate contacts with the LBD.

Elastic network model NMA has been applied much more extensively to large assemblies than all-atom MD simulations where the system size becomes an issue. Large conformational changes such as the splaying apart of the NTD dimers of iGluRs observed in low resolution electron microscopy studies [18,33] are beyond the scope of MD simulations; whereas they can be readily modelled by ENMs [94,99] (see Fig. 3, Fig. 4).

ANM analysis was initially applied to NTD monomers and dimers where clamshell closure and inter-subunit rearrangements were linked to allosteric signaling [26,93,94,100], and to an LBD tetramer [101]. The latter study revealed that the softest mode intrinsically accessible to the LBD tetramer is a cooperative rolling of the two dimers towards and away from each other that occurs during activation [101]. This cooperative motion has since been characterized further in the context of whole receptors both in structural studies [31] and using the ANM [99] as described next.

ANM studies of whole receptors provided a first glimpse of the dynamic coupling between the NTD, LBD and TMD layers [94,99,102]. These analyses further demonstrated the existence of cooperative modes of motions that are shared between AMPARs (exemplified by those sampled by the resting state GluA2 homomer) and NMDARs (exemplified by the ifenprodil-inhibited GluN1/N2B heteromer), as well as subtype-specific differences [94,99]. For example, the most energetically favorable mode (mode 1) is a global bending in both cases, with the NTD bending towards the TMD (or towards the plasma membrane, in a cellular context, into proximity of auxiliary subunits [36,38,103]), facilitated by the unstructured (∼16 aa) NTD-LBD linker [103]. In this mode, the loosely packed NTD-LBD interface acts as the main hinge region in the AMPAR whereas this region is tightly packed in the NMDAR so the LBD-TMD linker region is its main hinge site. Higher frequency modes showed more local motions such as a pivoting at the interface between NTD dimers coupled to rotations of the other domains and the aforementioned rolling apart of the LBD dimers [99] as well as clamshell motions of individual NTDs [94]. Iris-like rotations of the TMD were also seen reminiscent of channel opening. These motions were also seen in the presence of a lipid bilayer (also modelled using an ENM) which suppressed some modes and favored others [99].

A recent study by Popescu and coworkers used ENMs for flexible fitting of electron microscopy data and transition pathway modelling, yielding insights into NMDAR activation [104]. In addition to agreeing with conclusions from the original rigid-body fitting of isolated domains and portions thereof such as an opening of the NTD clefts and separation of the NTD dimers [27], the NMA-based flexible fitting revealed rearrangements of the LBD-TMD linkers consistent with pulling open the pore [104], but the data for the TMD was insufficient to enable resolution of the channel itself. This study further confirmed that two individual normal modes overlap well with the transition from the allosterically inhibited state to the active state [104]: both modes feature a separation of the two NTD dimers coupled to rotations of the other two domain layers akin to modes of motion observed in the earlier ANM study of whole AMPARs and NMDARs [99]. More detailed analysis of the transition with the interpolated ENM approach revealed sequential motion of the domains starting with the NTD relieving inhibition, followed by the NTD-LBD interface then the LBD itself, passed on to the LBD-TMD linkers and finally to the TMD.

## 5 Emerging mechanism of function of iGluRs from integration of ENM and MD data

iGluRs have a large extracellular region that facilitates an extensive spectrum of dynamic rearrangements, which are being extensively explored in structural and computational studies. All these observations translate into a *selective excitation of pre-existing motions* i.e. glutamate binding to the LBD selectively triggers cooperative motions from amongst those soft modes already encoded by the receptor structure. Such intrinsic modes of motion of whole receptors and isolated domains have been visualized using MD simulations [86,87,93,98] and ENM NMA [94,99,104], providing a deeper understanding of the relevance of particular conformational changes to activation, desensitization and allosteric modulation than that provided by the structures alone. Our ANM analysis of the intact AMPAR and NMDAR structures [94,99,102] revealed two cooperative global motions of particular interest, referred to as mode 4 and mode 6. These rearrangements appear to directly drive channel operation and likely influence binding to synaptic interaction partners to indirectly modulate receptor localization and signaling [7,8].

Mode 4 of the AMPAR (Fig. 3 and Movie 1) shows a good example of how the structural changes are triggered between domains. Coupled dynamics of the NTD and LBD induces contraction of the receptor with these domains coming towards each other and the LBD approaching the TMD [99], forming a compact structure that has been trapped in a cryo-EM study and also appears to be an inactive conformation [102]. Similar movements have been observed upon comparison of kainate/(R,R)-2b and ZK200775-bound states of AMPA receptors using ANM modes in a dynamic alignment scheme [105]. Interestingly, the compact structure (Fig. 3A) resembles the allosterically inhibited NMDAR (Fig. 3B), suggesting that this mode enables the structural transition related to entry into and release from an allosterically inhibited state as seen by following the mode in the other direction (see Fig. 3D). Associated with this is a change in the relative positioning of the NTD pairs of dimers (*bottom diagrams* and Movie 1), which vary from an O-shape organization in the compact state (panel A) through the classical N-shape in the starting structure (panel C) to a splayed apart, stretched shape at the other end of the mode (panel D).

The rearrangement of the NTDs in the compact AMPAR (panel A) approximates that of the inhibited NMDAR (panel B). The compact, inhibited-like state exhibits a close packing at the LBD-TMD interface where closure of the LBD clamshells could have less influence through the coiled LBD-TMD linkers not receiving as much tension. The release transition along this AMPAR ANM mode (Fig. 3C–D) shows the two NTD dimers separating from each other and from the LBD akin to transitions seen by comparing cryo-EM structures of NMDARs [27]. This collective motion also resembles conformational changes linked to desensitization in AMPARs [18,33]. Hence, it provides a possible explanation for the relationship between allosteric inhibition and desensitization in NMDARs [106] that may also hold in AMPARs.

The second global mode, mode 6 of the AMPAR, involves a set of cooperative dynamics, which is likely important for activation (Fig. 4A–B and Movie 2). In this case, we observe coupled inter-dimer pivoting motions in the LBD and NTD layers that are coupled to an iris-like rotation and opening of the TMD channel. These motions of the LBD and NTD have been confirmed by disulfide crosslinking [99,101] and are seen in structural studies that could not resolve channel opening [30,31,33,36,38,101,102]. The NTD motion was not captured in recent cryo-EM structures that resolved an open state of AMPAR in the presence of TARPs, possibly because one study used a truncated NTD-LBD linker (that aided crystallization by restricting NTD dynamics) [38], and the other masked out the NTD to improve resolution of the LBD and TMD sectors [36]. The rest of the motion closely approximates the structural change observed in the transition to the active structure (considering only the LBD and TMD). Thus, the ANM analysis allows us to infer a mechanism for activation that incorporates motions of all the domains that have been seen in separate structural studies: glutamate binding favors closure of individual LBDs [65,88], which pulls on inter-LBD interfaces, resulting in rearrangements such as a rolling of the dimers towards and away from each other pivoted about interfacial helix G, and a sliding of these helices past each other, that increase the pulling force on the other domains resulting in closure of the NTD layer and opening of the TMD channel pore. For a visualization of these cooperative events see Movie 2, where the motions of the intact AMPAR (side view) and those of the individual domains (cross-sectional views) can be clearly seen. The LBD D1 dimer interface is fairly stable on the microsecond timescale [92], allowing the above activation-related inter-dimer rearrangements to occur before the LBD dimer interface breaks and desensitization sets in.

Auxiliary subunits are optimally placed in close proximity to the LBD and TMD (Fig. 4C) to modulate this behavior and hence channel gating and ion conduction. For example, TARPs are known to favor channel opening and stabilize the activated state [8,36,38] whereas GSG1L disfavors channel opening and stabilizes inactive/desensitized states [36,37]. These functions may be achieved through the auxiliary subunits acting to constrain or amplify the intrinsically accessible cooperative motions related to TMD channel opening and desensitization.

There are still a number of unanswered questions that are likely to be addressed over the next few years through a combination of experimental and computational approaches. While key states and transitions can now be visualized, we are still lacking the energy landscape and a complete structural view of the gating cycle. It is also still unclear how auxiliary subunits and allosteric ligands modulate iGluRs. Recent and continuing developments in cryo-EM as well as hybrid simulation methods that benefit from the advantages of both NMA and MD are expected to shed light to these pending questions.

## 6 Conclusion

iGluRs undergo complex dynamics related to their functions, which are starting to be understood using experimental and computational studies. Two main types of computational methods have been applied that have their own strengths and limitations. All-atom MD simulations use detailed force fields that allow for analyzing and generating free energy landscapes but this comes at great computational cost that prevents the examination of cooperative events in large structures such as the multimeric iGluRs and the attainment of biologically relevant time scales. Coarse-grained representations such as ENMs, on the other hand, enable identification of biologically relevant global (soft) modes of motion that often agree with and build upon experimental data from structural studies, albeit at low resolution. In particular, these intrinsically accessible and energetically favorable modes of motion, analyzed in the context of existing experimental (structural) data and atomic-simulations performed for substructures, reveal well-defined couplings between the motions of AMPAR domains, and provide better glimpses of channel activation, desensitization and inhibition mechanisms than static structures alone. An emerging picture is an allosteric communication between the NTD, LBD and TMD modulated by glutamate binding to the LBD, which selectively triggers pre-existing robust modes uniquely encoded by the overall tetrameric architecture.

## Declarations of interest

None.

## References

[1] Sudhof T.C. (2017). Molecular neuroscience in the 21 st century: a personal perspective. Neuron.

[2] Traynelis S.F., Wollmuth L.P., McBain C.J., Menniti F.S., Vance K.M., Ogden K.K., Hansen K.B., Yuan H., Myers S.J., Dingledine R., Sibley D. (2010). Glutamate receptor ion channels: structure, regulation, and function. Pharmacol. Rev..

[3] Paoletti P., Bellone C., Zhou Q. (2013). NMDA receptor subunit diversity: impact on receptor properties, synaptic plasticity and disease. Nat. Rev. Neurosci..

[4] Jonas P. (2000). The time course of signaling at central glutamatergic synapses. News Physiol. Sci..

[5] Jackson A.C., Nicoll R.A. (2011). The expanding social network of ionotropic glutamate receptors: tARPs and other transmembrane auxiliary subunits. Neuron.

[6] Huganir R.L., Nicoll R.A. (2013). AMPARs and synaptic plasticity: the last 25 years. Neuron.

[7] Garcia-Nafria J., Herguedas B., Watson J.F., Greger I.H. (2016). The dynamic AMPA receptor extracellular region: a platform for synaptic protein interactions. J. Physiol..

[8] Greger I.H., Watson J.F., Cull-Candy S.G. (2017). Structural and functional architecture of AMPA-Type glutamate receptors and their auxiliary proteins. Neuron.

[9] Shepherd J.D., Huganir R.L. (2007). The cell biology of synaptic plasticity: AMPA receptor trafficking. Annu. Rev. Cell Dev. Biol..

[10] Kumar J., Mayer M.L. (2012). Functional insights from glutamate receptor ion channel structures. Annu. Rev. Physiol..

[11] Armstrong N., Gouaux E. (2000). Mechanisms for activation and antagonism of an AMPA-sensitive glutamate receptor: crystal structures of the GluR2 ligand binding core. Neuron.

[12] Armstrong N., Sun Y., Chen G.Q., Gouaux E. (1998). Structure of a glutamate-receptor ligand-binding core in complex with kainate. Nature.

[13] Kuusinen A., Abele R., Madden D.R., Keinanen K. (1999). Oligomerization and ligand-binding properties of the ectodomain of the alpha-amino-3-hydroxy-5-methyl-4-isoxazole propionic acid receptor subunit GluRD. J. Biol. Chem..

[14] Rosenmund C., Stern-Bach Y., Stevens C.F. (1998). The tetrameric structure of a glutamate receptor channel. Science.

[15] Stern-Bach Y., Bettler B., Hartley M., Sheppard P.O., O'Hara P.J., Heinemann S.F. (1994). Agonist selectivity of glutamate receptors is specified by two domains structurally related to bacterial amino acid-binding proteins. Neuron.

[16] Mayer M.L. (2006). Glutamate receptors at atomic resolution. Nature.

[17] Armstrong N., Jasti J., Beich-Frandsen M., Gouaux E. (2006). Measurement of conformational changes accompanying desensitization in an ionotropic glutamate receptor. Cell.

[18] Nakagawa T., Cheng Y., Ramm E., Sheng M., Walz T. (2005). Structure and different conformational states of native AMPA receptor complexes. Nature.

[19] Nakagawa T., Cheng Y., Sheng M., Walz T. (2006). Three-dimensional structure of an AMPA receptor without associated stargazin/TARP proteins. Biol. Chem..

[20] Sobolevsky A.I., Rosconi M.P., Gouaux E. (2009). X-ray structure, symmetry and mechanism of an AMPA-subtype glutamate receptor. Nature.

[21] Tichelaar W., Safferling M., Keinanen K., Stark H., Madden D.R. (2004). The three-dimensional structure of an ionotropic glutamate receptor reveals a dimer-of-dimers assembly. J. Mol. Biol..

[22] Clayton A., Siebold C., Gilbert R.J., Sutton G.C., Harlos K., McIlhinney R.A., Jones E.Y., Aricescu A.R. (2009). Crystal structure of the GluR2 amino-terminal domain provides insights into the architecture and assembly of ionotropic glutamate receptors. J. Mol. Biol..

[23] Jin R., Singh S.K., Gu S., Furukawa H., Sobolevsky A.I., Zhou J., Jin Y., Gouaux E. (2009). Crystal structure and association behaviour of the GluR2 amino-terminal domain. EMBO J..

[24] Karakas E., Simorowski N., Furukawa H. (2011). Subunit arrangement and phenylethanolamine binding in GluN1/GluN2B NMDA receptors. Nature.

[25] Rossmann M., Sukumaran M., Penn A.C., Veprintsev D.B., Babu M.M., Greger I.H. (2011). Subunit-selective N-terminal domain associations organize the formation of AMPA receptor heteromers. EMBO J..

[26] Sukumaran M., Rossmann M., Shrivastava I., Dutta A., Bahar I., Greger I.H. (2011). Dynamics and allosteric potential of the AMPA receptor N-terminal domain. EMBO J..

[27] Tajima N., Karakas E., Grant T., Simorowski N., Diaz-Avalos R., Grigorieff N., Furukawa H. (2016). Activation of NMDA receptors and the mechanism of inhibition by ifenprodil. Nature.

[28] Paoletti P., Perin-Dureau F., Fayyazuddin A., Le Goff A., Callebaut I., Neyton J. (2000). Molecular organization of a zinc binding N-Terminal modulatory domain in a NMDA receptor subunit. Neuron.

[29] Zhu S., Paoletti P. (2015). Allosteric modulators of NMDA receptors: multiple sites and mechanisms. Curr. Opin. Pharmacol..

[30] Chen L., Durr K.L., Gouaux E. (2014). X-ray structures of AMPA receptor-cone snail toxin complexes illuminate activation mechanism. Science.

[31] Durr K.L., Chen L., Stein R.A., De Zorzi R., Folea I.M., Walz T., McHaourab H.S., Gouaux E. (2014). Structure and dynamics of AMPA receptor GluA2 in resting, pre-open, and desensitized states. Cell.

[32] Yelshanskaya M.V., Li M., Sobolevsky A.I. (2014). Structure of an agonist-bound ionotropic glutamate receptor. Science.

[33] Meyerson J.R., Kumar J., Chittori S., Rao P., Pierson J., Bartesaghi A., Mayer M.L., Subramaniam S. (2014). Structural mechanism of glutamate receptor activation and desensitization. Nature.

[34] Twomey E.C., Yelshanskaya M.V., Grassucci R.A., Frank J., Sobolevsky A.I. (2016). Elucidation of AMPA receptor-stargazin complexes by cryo-electron microscopy. Science.

[35] Zhao Y., Chen S., Yoshioka C., Baconguis I., Gouaux E. (2016). Architecture of fully occupied GluA2 AMPA receptor-TARP complex elucidated by cryo-EM. Nature.

[36] Chen S., Zhao Y., Wang Y., Shekhar M., Tajkhorshid E., Gouaux E. (2017). Activation and desensitization mechanism of AMPA receptor-TARP complex by cryo-EM. Cell.

[37] Twomey E.C., Yelshanskaya M.V., Grassucci R.A., Frank J., Sobolevsky A.I. (2017). Structural bases of desensitization in AMPA receptor-auxiliary subunit complexes. Neuron.

[38] Twomey E.C., Yelshanskaya M.V., Grassucci R.A., Frank J., Sobolevsky A.I. (2017). Channel opening and gating mechanism in AMPA-subtype glutamate receptors. Nature.

[39] Karakas E., Furukawa H. (2014). Crystal structure of a heterotetrameric NMDA receptor ion channel. Science.

[40] Lee C.H., Lu W., Michel J.C., Goehring A., Du J., Song X., Gouaux E. (2014). NMDA receptor structures reveal subunit arrangement and pore architecture. Nature.

[41] Zhu S., Stein R.A., Yoshioka C., Lee C.H., Goehring A., McHaourab H.S., Gouaux E. (2016). Mechanism of NMDA receptor inhibition and activation. Cell.

[42] Mayer M.L. (2017). The Challenge of Interpreting Glutamate-Receptor Ion-Channel Structures.

[43] Cooper D.R., Dolino D.M., Jaurich H., Shuang B., Ramaswamy S., Nurik C.E., Chen J., Jayaraman V., Landes C.F. (2015). Conformational transitions in the glycine-bound GluN1 NMDA receptor LBD via single-molecule FRET. Biophys. J ..

[44] Dolino D.M., Cooper D., Ramaswamy S., Jaurich H., Landes C.F., Jayaraman V. (2015). Structural dynamics of the glycine-binding domain of the N-methyl-d-aspartate receptor. J. Biol. Chem..

[45] Dolino D.M., Rezaei Adariani S., Shaikh S.A., Jayaraman V., Sanabria H. (2016). conformational selection and submillisecond dynamics of the ligand-binding domain of the N-methyl-d-aspartate receptor. J. Biol. Chem..

[46] Du M., Rambhadran A., Jayaraman V. (2008). Luminescence resonance energy transfer investigation of conformational changes in the ligand binding domain of a kainate receptor. J. Biol. Chem..

[47] Gonzalez J., Du M., Parameshwaran K., Suppiramaniam V., Jayaraman V. (2010). Role of dimer interface in activation and desensitization in AMPA receptors. Proc. Natl. Acad. Sci. U. S. A..

[48] Gonzalez J., Rambhadran A., Du M., Jayaraman V. (2008). LRET investigations of conformational changes in the ligand binding domain of a functional AMPA receptor. Biochemistry.

[49] Landes C.F., Rambhadran A., Taylor J.N., Salatan F., Jayaraman V. (2011). Structural landscape of isolated agonist-binding domains from single AMPA receptors. Nat. Chem. Biol..

[50] Ramaswamy S., Cooper D., Poddar N., MacLean D.M., Rambhadran A., Taylor J.N., Uhm H., Landes C.F., Jayaraman V. (2012). Role of conformational dynamics in alpha-amino-3-hydroxy-5-methylisoxazole-4-propionic acid (AMPA) receptor partial agonism. J. Biol. Chem..

[51] Rambhadran A., Gonzalez J., Jayaraman V. (2011). Conformational changes at the agonist binding domain of the N-methyl-d-aspartic acid receptor. J. Biol. Chem..

[52] Shaikh S.A., Dolino D.M., Lee G., Chatterjee S., MacLean D.M., Flatebo C., Landes C.F., Jayaraman V. (2016). Stargazin modulation of AMPA receptors. Cell Rep..

[53] Sirrieh R.E., MacLean D.M., Jayaraman V. (2013). Amino-terminal domain tetramer organization and structural effects of zinc binding in the N-methyl-d-aspartate (NMDA) receptor. J. Biol. Chem..

[54] Sirrieh R.E., MacLean D.M., Jayaraman V. (2015). A conserved structural mechanism of NMDA receptor inhibition: a comparison of ifenprodil and zinc. J. Gen. Physiol..

[55] Sirrieh R.E., MacLean D.M., Jayaraman V. (2015). Subtype-dependent N-methyl-d-aspartate receptor amino-terminal domain conformations and modulation by spermine. J. Biol. Chem..

[56] Bahar I., Cheng M.H., Lee J.Y., Kaya C., Zhang S. (2015). Structure-encoded global motions and their role in mediating protein-substrate interactions. Biophys. J..

[57] Bahar I., Lezon T.R., Yang L.W., Eyal E. (2010). Global dynamics of proteins: bridging between structure and function. Annu. Rev. Biophys..

[58] Wei G., Xi W., Nussinov R., Ma B. (2016). Protein ensembles how does nature harness thermodynamic fluctuations for life? The diverse functional roles of conformational ensembles in the cell. Chem. Rev..

[59] Adcock S.A., McCammon J.A. (2006). Molecular dynamics: survey of methods for simulating the activity of proteins. Chem. Rev..

[60] Eswar N., Webb B., Marti-Renom M.A., Madhusudhan M.S., Eramian D., Shen M.Y., Pieper U., Sali A. (2007). Comparative protein structure modeling using MODELLER. Curr. Protoc. Protein Sci..

[61] Sali A., Blundell T.L. (1993). Comparative protein modelling by satisfaction of spatial restraints. J. Mol. Biol..

[62] Vanommeslaeghe K., MacKerell A.D. (2015). CHARMM additive and polarizable force fields for biophysics and computer-aided drug design. Biochim. Biophys. Acta.

[63] Shaw D.E., Dror R.O., Salmon J.K., Grossman J.P., Mackenzie K.M., Bank J.A., Young C., Deneroff M.M., Batson B., Bowers K.J., Chow E., Eastwood M.P., Ierardi D.J., Klepeis J.L., Kuskin J.S., Larson R.H., Lindorff-Larsen K., Maragakis P., Moraes M.A., Piana S., Shan Y., Towles B. (2009). Millisecond-scale molecular dynamics simulations on Anton. Proceedings of the Conference on High Performance Computing Networking, Storage and Analysis.

[64] Bernardi R.C., Melo M.C., Schulten K. (2015). Enhanced sampling techniques in molecular dynamics simulations of biological systems. Biochim. Biophys. Acta.

[65] Lau A.Y., Roux B. (2007). The free energy landscapes governing conformational changes in a glutamate receptor ligand-binding domain. Structure.

[66] Mamonova T., Yonkunas M.J., Kurnikova M.G. (2008). Energetics of the cleft closing transition and the role of electrostatic interactions in conformational rearrangements of the glutamate receptor ligand binding domain. Biochemistry.

[67] Bahar I., Lezon T.R., Bakan A., Shrivastava I.H. (2010). Normal mode analysis of biomolecular structures: functional mechanisms of membrane proteins. Chem. Rev..

[68] Atilgan A.R., Durell S.R., Jernigan R.L., Demirel M.C., Keskin O., Bahar I. (2001). Anisotropy of fluctuation dynamics of proteins with an elastic network model. Biophys. J..

[69] Doruker P., Atilgan A.R., Bahar I. (2000). Dynamics of proteins predicted by molecular dynamics simulations and analytical approaches: application to alpha-amylase inhibitor. Proteins.

[70] Eyal E., Lum G., Bahar I. (2015). The anisotropic network model web server at 2015 (ANM 2.0). Bioinformatics.

[71] Gur M., Zomot E., Bahar I. (2013). Global motions exhibited by proteins in micro- to milliseconds simulations concur with anisotropic network model predictions. J. Chem. Phys..

[72] Atilgan C., Atilgan A.R. (2009). Perturbation-response scanning reveals ligand entry-exit mechanisms of ferric binding protein. PLoS Comput. Biol..

[73] Chennubhotla C., Bahar I. (2007). Signal propagation in proteins and relation to equilibrium fluctuations. PLoS Comput. Biol..

[74] Bahar I., Atilgan A.R., Demirel M.C., Erman B. (1998). Vibrational dynamics of folded proteins: significance of slow and fast motions in relation to function and stability. Phys. Rev. Lett..

[75] Bahar I., Atilgan A.R., Erman B. (1997). Direct evaluation of thermal fluctuations in proteins using a single-parameter harmonic potential. Fold. Des..

[76] Haliloglu T., Bahar I., Erman B. (1997). Gaussian dynamics of folded proteins. Phys. Rev. Lett..

[77] Li H., Chang Y.Y., Lee J.Y., Bahar I., Yang L.W. (2017). DynOmics: dynamics of structural proteome and beyond. Nucleic Acids Res..

[78] Armstrong N., Sun Y., Chen G.Q., Gouaux E. (1998). Structure of a glutamate-receptor ligand-binding core in complex with kainate. Nature.

[79] Arinaminpathy Y., Sansom M.S., Biggin P.C. (2002). Molecular dynamics simulations of the ligand-binding domain of the ionotropic glutamate receptor GluR2. Biophys. J..

[80] Speranskiy K., Kurnikova M. (2005). On the binding determinants of the glutamate agonist with the glutamate receptor ligand binding domain. Biochemistry.

[81] Kubo M., Odai K., Sugimoto T., Ito E. (2001). Quantum chemical study of agonist-receptor vibrational interactions for activation of the glutamate receptor. J. Biochem..

[82] Kubo M., Shiomitsu E., Odai K., Sugimoto T., Suzuki H., Ito E. (2003). Agonist-specific vibrational excitation of glutamate receptor. J. Mol. Struct.: Theochem..

[83] Kubo M., Shiomitsu E., Odai K., Sugimoto T., Suzuki H., Ito E. (2004). Picosecond dynamics of the glutamate receptor in response to agonist-induced vibrational excitation. Proteins.

[84] Mendieta J., Gago F., Ramirez G. (2005). Binding of 5'-GMP to the GluR2 AMPA receptor: insight from targeted molecular dynamics simulations. Biochemistry.

[85] Mendieta J., Ramirez G., Gago F. (2001). Molecular dynamics simulations of the conformational changes of the glutamate receptor ligand-binding core in the presence of glutamate and kainate. Proteins.

[86] Arinaminpathy Y., Sansom M.S., Biggin P.C. (2006). Binding site flexibility: molecular simulation of partial and full agonists within a glutamate receptor. Mol. Pharmacol..

[87] Kaye S.L., Sansom M.S., Biggin P.C. (2006). Molecular dynamics simulations of the ligand-binding domain of an N-methyl-d-aspartate receptor. J. Biol. Chem..

[88] Lau A.Y., Roux B. (2011). The hidden energetics of ligand binding and activation in a glutamate receptor. Nat. Struct. Mol. Biol..

[89] Yao Y., Belcher J., Berger A.J., Mayer M.L., Lau A.Y. (2013). Conformational analysis of NMDA receptor GluN1, GluN2, and GluN3 ligand-binding domains reveals subtype-specific characteristics. Structure.

[90] Yu A., Alberstein R., Thomas A., Zimmet A., Grey R., Mayer M.L., Lau A.Y. (2016). Molecular lock regulates binding of glycine to a primitive NMDA receptor. Proc. Natl. Acad. Sci. U. S. A..

[91] Musgaard M., Biggin P.C. (2016). Steered molecular dynamics simulations predict conformational stability of glutamate receptors. J. Chem. Inf. Model.

[92] Yonkunas M., Buddhadev M., Flores Canales J.C., Kurnikova M.G. (2017). Configurational preference of the glutamate receptor ligand binding domain dimers. Biophys. J..

[93] Dutta A., Shrivastava I.H., Sukumaran M., Greger I.H., Bahar I. (2012). Comparative dynamics of NMDA- and AMPA-Glutamate receptor N-Terminal domains. Structure.

[94] Krieger J., Bahar I., Greger I.H. (2015). Structure, dynamics, and allosteric potential of ionotropic glutamate receptor N-Terminal domains. Biophys. J..

[95] Dong H., Zhou H.X. (2011). Atomistic mechanism for the activation and desensitization of an AMPA-subtype glutamate receptor. Nat. Commun..

[96] Dai J., Zhou H.X. (2013). An NMDA receptor gating mechanism developed from MD simulations reveals molecular details underlying subunit-specific contributions. Biophys. J..

[97] Kazi R., Dai J., Sweeney C., Zhou H.X., Wollmuth L.P. (2014). Mechanical coupling maintains the fidelity of NMDA receptor-mediated currents. Nat. Neurosci..

[98] Yu A., Lau A.Y. (2017). Energetics of glutamate binding to an ionotropic glutamate receptor. J. Phys. Chem. B.

[99] Dutta A., Krieger J., Garcia-Nafria J., Lee J., Greger I.H., Bahar I. (2015). Cooperative dynamics in intact AMPA and NMDA glutamate receptors –similarities and subfamily-specific differences. Structure.

[100] Zhu S., Stroebel D., Yao C.A., Taly A., Paoletti P. (2013). Allosteric signaling and dynamics of the clamshell-like NMDA receptor GluN1 N-terminal domain. Nat. Struct. Mol. Biol..

[101] Lau A.Y., Salazar H., Blachowicz L., Ghisi V., Plested A.J., Roux B. (2013). A conformational intermediate in glutamate receptor activation. Neuron.

[102] Herguedas B., Garcia-Nafria J., Cais O., Fernandez-Leiro R., Krieger J., Ho H., Greger I.H. (2016). Structure and organization of heteromeric AMPA-type glutamate receptors. Science.

[103] Cais O., Herguedas B., Krol K., Cull-Candy S.G., Farrant M., Greger I.H. (2014). Mapping the interaction sites between AMPA receptors and TARPs reveals a role for the receptor N-terminal domain in channel gating. Cell Rep..

[104] Zheng W., Wen H., Iacobucci G.J., Popescu G.K. (2017). Probing the structural dynamics of the NMDA receptor activation by coarse-grained modeling. Biophys. J..

[105] Tobi D. (2017). Dynamical differences of hemoglobin and the ionotropic glutamate receptor in different states revealed by a new dynamics alignment method. Proteins.

[106] Erreger K., Traynelis S.F. (2005). Allosteric interaction between zinc and glutamate binding domains on NR2A causes desensitization of NMDA receptors. J. Physiol..

